# Differential analysis of free and bound phenolic compounds and its antioxidant activity of *Ficus hirta* Vahl. root cortex with different leaf morphotypes based on metabolomics

**DOI:** 10.1016/j.fochx.2026.103554

**Published:** 2026-01-25

**Authors:** Qing Gui, Qingmian Chen, Yufeng Zhang, Xiu Zeng, Shiyu Li, Jianxiong Huang, Xiuquan Wang

**Affiliations:** a Rubber Research Institute of Chinese Academy of Tropical Agricultural Sciences, Haikou 570100, China; b Institute of Environmental and Plant Protection, Chinese Academy of Tropical Agricultural Science, Haikou 571101, China; c Coconut Research Institute of Chinese Academy of Tropical Agricultural Sciences, Wenchang 571339, China; d College of Tropical Crop Science, Yunnan Agricultural University, Pu'er 665099, China

**Keywords:** Free phenolics, Bound phenolics, Leaf morphotypes, Quantitative analysis

## Abstract

The free and bound phenolic compounds and its antioxidant activities in the root cortext of *F. hirta* Vahl. were analyzed by UPLC-MS. 57 phenolic compounds were identified, among which 49 are novel and reported for the first time. Psoralen was the most common free phenolic, followed by coniferin and bergapten. Coniferin, afzelechin, naringenin, ferulic acid, and umbelliferone were the predominant bound phenolics. Both free and bound phenolic fraction exhibited relatively high capacity to scavenge DPPH free radicals. According to PCA and HCA analysis, free phenolic fraction in five-lobed leaf type were distinct from unlobed and three-lobed. Pearson correlation identified that psoralen and other 18 phenolic compounds were positively correlated with antioxidant activity. These findings can provide theoretical basis for the breeding of *F. hirta* Vahl. varieties to maximize phenol production for later development and commercial utilization.

## Highlights

• 49 phenolic compounds were quantified for the first time.
• Psoralen, bergapten and coniferin were the most abundant phenolics.
• Psoralen were positively correlated with antioxidant activity.
• Free phenolics fraction of FL were distinct from NL and TL.

## 1 Introduction

*Ficus hirta* Vahl. (Moraceae), a dual-purpose medicinal and edible plant distributed widely across southern China and Southeast Asia, is also known as “Guangdong ginseng” and is traditionally used both in Hakka cuisine and for herbal medicine (Au et al., 2008). The roots of *F. hirta* Vahl. are rich in flavonoids, coumarins, and polysaccharides, considered medicinal for immunomodulatory, anti-inflammatory, and hepatoprotective effects, and there are emerging applications for the commercial production of these compounds for the treatment of metabolic disorders and viral infections (e.g., SARS-CoV-2) (Chen, Tan, et al., 2021). Notably, aqueous extracts of *F. hirta* Vahl. roots demonstrate therapeutic potential against tumors, chronic hepatitis, and gut microbiota dysregulation, attributed to their antioxidant capacity and regulation of lipid metabolism (Cheng, Yi, Chen, et al., 2017; Quan et al., 2022; Xiao et al., 2022). Phenolic compounds, secondary metabolites in *F. hirta* Vahl. roots, exhibit both anti-neuroinflammatory and anticancer properties‌ (Chen et al., 2020; Yang et al., 2018; Ye et al., 2020). Current studies primarily focus on ethanol-extracted free phenolics (Cheng, Yi, Chen, et al., 2017; Cheng, Yi, Wang, et al., 2017; Ye et al., 2020). However, bound phenolics conjugated to cell wall components (e.g., cellulose, lignin) remain underexplored despite constituting >50% of total phenolic content in plants and increasing evidence bound molecules, although less bioactive, may offer colorectal health benefits through slow, prolonged release (Zeng et al., 2023). This omission of bound phenol characterization consequently limits a comprehensive understanding of their synergistic roles in disease prevention and functional food development (Acosta-Estrada et al., 2014; Fernandes et al., 2023; Su et al., 2014). Our previous studies have shown that polyphenols were the most abundant active ingredient in *F. hirta* Vahl. and mainly present in the root cortex (Gui et al., 2023).

Previous studies have shown that environment and genetic diversity may each influence both plant morphotype and chemotype, and that morphotypic diversity may be related to chemotypic diversity under certain conditions. The nutritional values of *H. sabdariffa* leaves could be predicted by morphotype (Sambou et al., 2023). It was reported that there were significant differences of triterpenic compounds in *A. millefolium* of different morphotypes, and that the morphotype may serve as a marker for the selection of a certain chemotype (Raudone et al., 2024). Plant phenotype was also related to active metabolites in *Acanthopanax senticosus* (Sun et al., 2012). If we did not specify the variety and the morphotype, it would be difficult to make comparisons between the chemical profile and bioactivities of extracts obtained in different studies (Borrás-Linares et al., 2015). Our field observations revealed that *F. hirta* Vahl. presents with different leaf morphotypes (unlobed, three-lobed, and five-lobed‌) under the same cultivation conditions. Traditional knowledge posited five-lobed roots as superior, yet scientific validation of leaf morphology-phytochemical correlations was lacking‌. We will incorporate this morphotypic designation in our study to better improve our understanding of *F. hirta* Vahl.

This study integrated‌ UHPLC-MS/MS based on metabolomics to quantify the contents of free and bound phenols in three different leaf morphotypes of *F. hirta* Vahl. root cortex, as well as the *in vitro* antioxidant activities. Further, the relationship between polyphenol and antioxidant activity was systematically studied by multivariate statistical analysis, and the key phenolic compounds related to antioxidant activity were identified. This provided theoretical support for breeding new varieties with high active ingredients and further development and utilization of *F. hirta* Vahl. root.

## 2 Materials and methods

### 2.1 Plant material

**Fig. 1:**
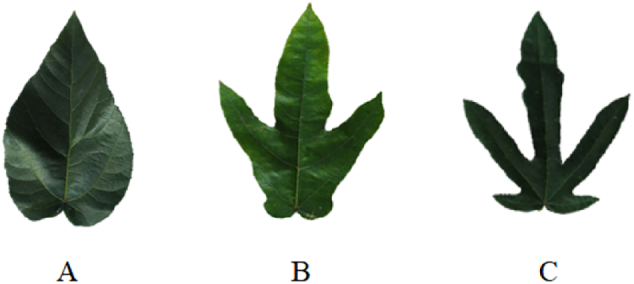
Leaf of *F. hirta* Vahl. (A) Leaf of *F. hirta* Vahl. with unlobed: NL; (B) Leaf of *F. hirta* Vahl. with three-lobed: TL; (C) Leaf of *F. hirta* Vahl. with five-lobed: FL.

Root samples of *F. hirta* Vahl. with three distinct leaf morphotypes NL (unlobed), TL (three-lobed)‌, and ‌FL (five-lobed) (Fig. 1) were collected from a rubber plantation (19°32′55″N, 109°28′30″E) managed by the Chinese Academy of Tropical Agricultural Sciences (CATAS) at November 2023. All root samples were planted for 2 years in the rubber plantation base. 15 stocks of each variety was collected from the same plot, meaning these plants were all exposed to the same planting environment and selected at the same harvest time. All roots‌ were cryopreserved at −80 °C immediately after harvest. Following collection, the ‌root cortex of distinct leaf morphotypes were peeled off. Afterwards, root cortex was dried in vacuum freeze dryer (scientz-30YG/A, China) at −20 °C to constant weight. Lyophilized samples were then crush into 100 mesh and stored in amber vials at −20 °C for further analysis.

### 2.2 Chemicals

Folin-Ciocalteu reagent, 1,1-diphenyl-2-picrylhydrazyl (DPPH), ascorbic acid, 2,2′-Azino-bis (3-ethylbenzthiazoline-6-sulfonic acid), and diammonium salt (ABTS) were purchased from Yuanye Biotechnology Co. Ltd. (Shanghai, China). All phenolic compounds standards were purchased from Aladdin Biotechnology Co. Ltd. LC/MS grade reagents were purchased from Thermo Fisher Scientific Co. (MA, USA). Sodium hydroxide, hydrochloric acid, sodium carbonate, ethanol, acetone, methanol, ferrous sulfate, hydrogen peroxide, salicylic acid, and dimethyl sulfoxide were obtained from China National Pharmaceutical Group Corporation (Beijing, China).

### 2.3 Extraction and preparation of free and bound phenolics

Free and bound phenolics were extracted according to a previous study (Govardhan et al., 2013; Irakli et al., 2012; Arruda et al., 2018; Wang et al., 2019; Yao et al., 2021). For free phenolics extraction, the sample powder was subjected to solvent optimization using 20%–100% (*V*/*V*) acetone, 80% (*V*/*V*) ethanol, 80% (*V*/*V*) methanol, methanol:acetone: water (*V:V:V* = 1:1:1). Following optimization, extraction was performed at at a ratio of 1:20 (m/*V*) and ultrasonicated (KQ-250DE, Kunshan, Jiangsu, China) at 40 KHz at room temperature for 30 min. The extracts were then centrifuged at 1500 ×*g* and 4 °C for 10 min (Heal force, Neofuge 15R, Shanghai, China) to obtain the liquid supernatant. The resulting pellet was subsequently extracted three times under ‌identical conditions. All supernatant was mixed, and evaporated under vacuum at 35 °C to remove the organic solvent. The concentrated solution was dissolved with methanol and filtered through a 0.22 μm filter, and labeled as free phenolic fraction (FP).

After extracting free phenolics, the residue was mixed with 4 mol/L sodium hydroxide at a ratio of 1:20 g/mL (m/V), and hydrolyzed at 70 °C for 3 h. After alkaline hydrolysis, the solution was acidified to pH 2.0 with 6 mol/L hydrochloric acid, and centrifuged at 1500 ×*g* and 4 °C for 10 min to remove precipitates. The supernatant was processed with 40 mL hexane to remove free fatty acids and other lipid contaminants. The aqueous layer was then extracted three times with ethyl acetate. Subsequently, ethyl acetate extracts were evaporated to dryness in a rotary evaporator (RE, 52 A, Shanghai, China) at 35 °C under vacuum. The dry residue was dissolved in methanol, filtered through a 0.22 μm filter, and then labeled as bound phenolic fraction (BP).

### 2.4 Determination of total polyphenol and total flavonoids content

The content of total phenolics of FP and BP were determined via the Folin-Ciocalteu reagent method (Yang et al., 2020). Briefly, 0.2 mL extract solution was mixed with 0.75 mL of Folin-Ciocalteu reagent. After stirring, 4.0 mL of 75 g/L Na_2_CO_3_ solution was added. After stirring, the mixture was kept in the dark for 1 h. Afterward, the absorbance was measured at 765 nm. The results were expressed as milligram of gallic acid equivalents (GAE) per gram of sample dry weight.

The content of total flavonoids of FP and BP was measured according to the modified method reported by Kang et al. (2010). 0.5 mL of 5% NaNO_2_ solution was added into 0.5 mL sample extract. After incubating for 6 min at room temperature, 0.5 mL of 10% AlCl_3_ solution was added. After another 5 min, 1.0 mL of 4% NaOH was added. The absorbance was immediately measured at 510 nm. Results were expressed as milligram of rutin equivalents (RE) per gram of sample dry weight.

### 2.5 Quantitative analysis of free and bound phenolics

After extraction and filtration of FP and BP, the extract solution was separated using a UHPLC system (Acquity UPLC H-Class, Waters Corporation, Milford, DE, USA) equipped with a C_18_ column (1.7 μm, 3.0 mm × 5 mm, BEH—C_18_, Waters Corporation, Milford, DE, USA). The mobile phase consisted of solvent A: pure water with 0.1% formic acid, and solvent B: acetonitrile. Gradient elution was performed following a previously reported procedure as follows (Tang et al., 2021): 0–0.5 min, 10% B; 0.5–15 min, 10–60% B; 15–16 min, 60–90%; 16–18 min, 90% B; 18–20 min, 95% B; 20-25 min, 10% B; 25-30 min, 10% B. The injection volume was 10 μL. The column temperature was set at 30 °C, and the flow rate was set at 0.2 mL/min.

The mass spectrogram was acquired in using an Xevo TQ-S micro mass spectrometer (Waters Corporation, Milford, DE, USA). Typical ion source parameters were as follows: ion spray voltage, +5500/− 4500 V; and temperature, 500 °C. For identifying phenolic compounds, *m*/*z* transitions of precursor and product ions were conducted under the multiple reactions monitoring (MRM) mode. Preliminary identification of these compounds was performed by comparing the retention times (Rt), interpreting the information on parent and daughter ions and by matching the above data with the concerned standards. The peak area and concentration of studied phenolic standards were plotted to establish a calibration curve, and the results were expressed as microgram of compound per gram of sample dry weight.

### 2.6 Antioxidant activity assays in vitro

The DPPH• scavenging abilities of FP and BP were determined according to Song et al. (2022). The ABTS• assay was conducted following established protocols (Werf et al., 2014). The hydroxyl radical (OH•) scavenging activity of extracts was assessed according to the method reported by Zhang et al. (Zhang et al., 2012). The half inhibitory concentration (IC_50_) of DPPH•, ABTS• and OH• values were expressed as microgram Vitamin C (Vc) equivalents per gram of sample.

### 2.7 Statistical analysis

All experiments were performed in triplicate and results were expressed as means ± standard deviation from three independent replicates. One-way ANOVA followed by Duncan's multiple-range test was used to assess the differences between the individual groups. Differences were considered significant when *p* *<* *0.05*. IC_50_ values were calculated by linear approximation regression. Principal component analysis (PCA), hierarchical clustering analysis (HCA) and pearson correlation analysis were performed using SPSS statistics 23 (IBM, Chicago, IL, USA), and graphs were generated using Microsoft Excel 2010 and Origin 2019.

## 3 Results and discussion

### 3.1 Effect of extraction solvents on the content of polyphenols

For most phenolics and flavanoids, the polar and medium polar solvents, such as water, ethanol, methanol, propanol, acetone and their aqueous mixtures, are widely used for extraction (Xu et al., 2017). Do et al. (2014) suggested that the presence of water would increase extraction yield of total phenol and total flavonoid content by enhancing cell wall penetration by the solvent. We found that a solution of 80% acetone content led to the highest extraction rate of total polyphenols from yuzu, as well as for buckwheat, 80% acetone, 80% methanol and 80% ethanol all exhibited comparable extraction efficiency for total polyphenols. (Assefa & Keum, 2017; Podloucká et al., 2025). Research by Wiyono et al. (2020) also showed that extracted polyphenol content increased with increasing ethanol levels (40%–80%) for a time, before reaching a saturation and eventually a decline in extraction when ethanol continues to be increased. Other studies have found a methanol/acetone/water (7:7:6, *V/V/V*) mixture may be used to obtain free, soluble-bound phenolics and insoluble-bound phenolics from raspberry pomace by Yao et al. (2021).

**Fig. 2:**
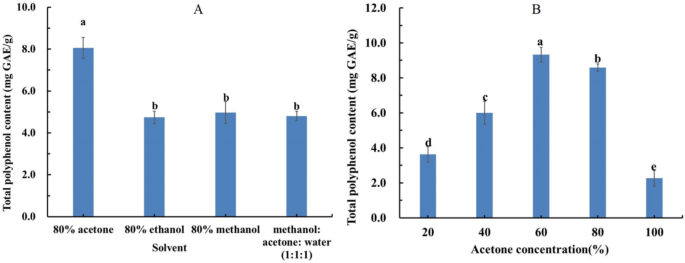
Total polyphenol content of *F. hirta* Vahl. root cortex obtained by various solvents. A: total polyphenol content of *F. hirta* Vahl. root cortex *F. hirta* Vahl. root cortex extracted by 80% acetone, 80% ethanol, 80% methanol and Methanol: Acetone: Water (V:V:V = 1:1:1);B: total polyphenol content of *F. hirta* Vahl. root cortex *F. hirta* Vahl. root cortex extracted by 20%, 40%, 60%,80% and 100% acetone. Different lowercase letters (a-c) indicated statistically significant differences between different solvents (*p* < 0.05).

Previous studies on polyphenol extraction from *F. hirta* Vahl. primarily employed ethanol aqueous solutions for extraction (Cheng, Yi, Chen, et al., 2017; Cheng, Yi, Wang, et al., 2017). However, our single-factor experiments shown in Fig. 2 A, suggested total polyphenol contents of *F. hirta* Vahl. root cortex extracted by 80% acetone was 8.06 ± 0.50 mg GAE/g, which was significantly higher than the samples extracted by solution of 80% ethanol, 80% methanol and a mixture solution of methanol: acetone: water (*V:V:V* = 1:1:1). We also observed 60% acetone (9.32 ± 0.42 mg GAE/g) had superior extraction of polyphenols compared to that of 80% acetone (Fig. 2 B). It was demonstrated that acetone may be effective in isolating polyphenol compounds in *F. hirta* Vahl. root cortex, which was consistent with the results for polyphenols extracted from *F. hirta* Vahl. fruit (Chen et al., 2020).

### 3.2 Toal polyphenol and flavonoids content of FP and BP

**Fig. 3:**
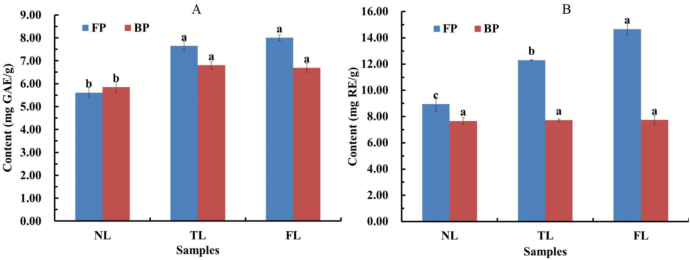
Total phenolic total flavonoid content in FP and BP of *F. hirta* Vahl. root cortex of three morphotypes: NL, TL and FL. A: total phenolic content of FP and BP; B: total flavonoid content of FP and BP. Different lowercase letters (a-c) indicated statistically significant differences between different leaf morphotypes (*p* < 0.05). The results of total phenolics were expressed as milligram of gallic acid equivalents (GAE) per gram of sample dry weight. Results of total flavonoids were expressed as milligram of rutin equivalents (RE) per gram of sample dry weight.

The FP and BP of *F. hirta* Vahl. root cortex of various morphotypes (NL, TL and FL) exhibited distinct profiles. Concentrations of total phenolics in FP were 5.61 ± 0.23 (NL), 7.64 ± 0.21 (TL), and 8.01 ± 0.13 (FL) mg GAE/g, of which the bound fractions measured 5.85 ± 0.24 (NL), 6.80 ± 0.16 (TL), and 6.69 ± 0.18 (FL)‌ mg GAE/g, respectively (Fig. 3 A). There were no significant differences in either free or bound phenolic concentration between TL and FL (*p* *>* *0.05*)‌, but they both were significantly higher than that of NL (*p* *<* *0.05*). Through alkaline hydrolysis, we revealed a previously overlooked reservoir of bound phenolic conjugates, accounting for 45.5–51.0% of total phenolic content. These findings establish a strategy for unlocking phytochemicals from other macromolecular substances in *F. hirta* Vahl.

The content of total flavonoids in FP and BP in *F. hirta* Vahl. root cortex of each distinct morphotype are presented in Fig. 3 B. Of the free phenol fraction, total flavonoids concentrations were 8.94 ± 0.55, 12.30 ± 0.05, 14.67 ± 0.48 mg RE/g, respectively, and each morphotype was distinct (*p* *<* *0.05*). Total flavonoids levels in the bound fraction ranged from 7.66 to 7.72 mg RE/g and did not vary among the morphotypes (*p* *>* *0.05*). This phenomenon might be attributed to interference events affecting bound flavonoids during sequential processing stages, specifically hydrolysis, pH, extraction and concentration (Wang, Sun, et al., 2016).

Previous studies have demonstrated differences in polyphenol content among various leaf morphotypes of leafy or inflorescence vegetables (Bonasia et al., 2013; Conversa, Bonasia, Lazzizera and Elia, 2016, Conversa, Lazzizera, Bonasia, La Rotonda and Elia, 2020). However, the differences in polyphenol content are related to multiple factors, such as variety, harvest time, cultivation conditions, etc. (Wang, Cao, et al., 2016; Ryu et al., 2016). These factors may obscure any difference we would have observed in this study. For example, no significant difference between polyphenol and tannin content between semi-palmate and palmate leaf of *H. sabdariffa*, probably due to overriding environmental factors (Sambou et al., 2023). Whether phenolics composition in *F. hirta* Vahl. root cortex is related to its leaf morphotype still requires further research.

### 3.3 Quantification analysis of individual polyphenols of FP and BP

**Fig. 4:**
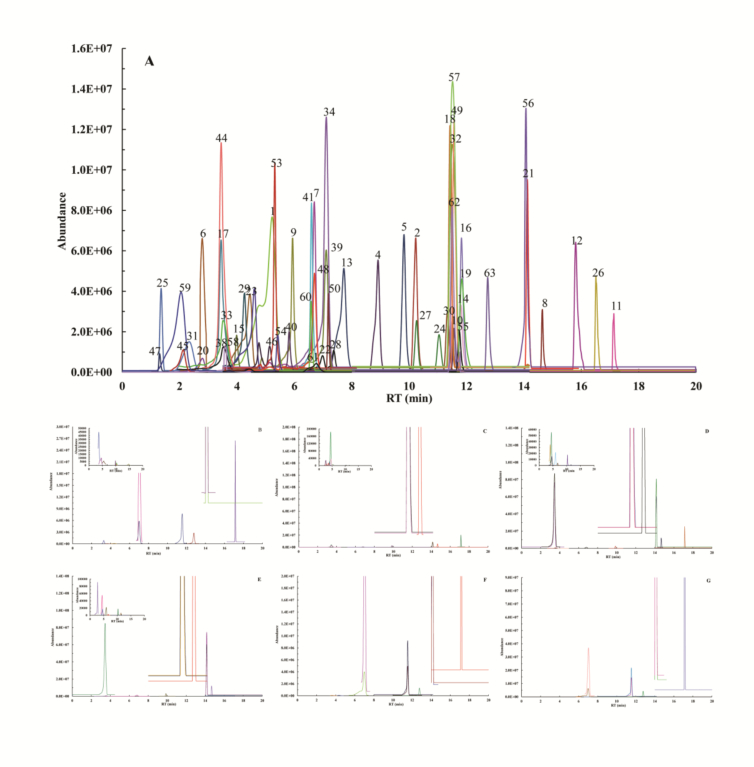
Extracted ion chromatogram (EIC) of 56 phenolic compounds of standard substance (A), free phenolic fractions of NL (B), TL (C) and FL (D), bound phenolic fractions of NL (E), TL (F) and FL (G) on positive mode. Compounds are numbered based on retention times (RT) and the corresponding names are given in Supplementary documents.

**Fig. 5:**
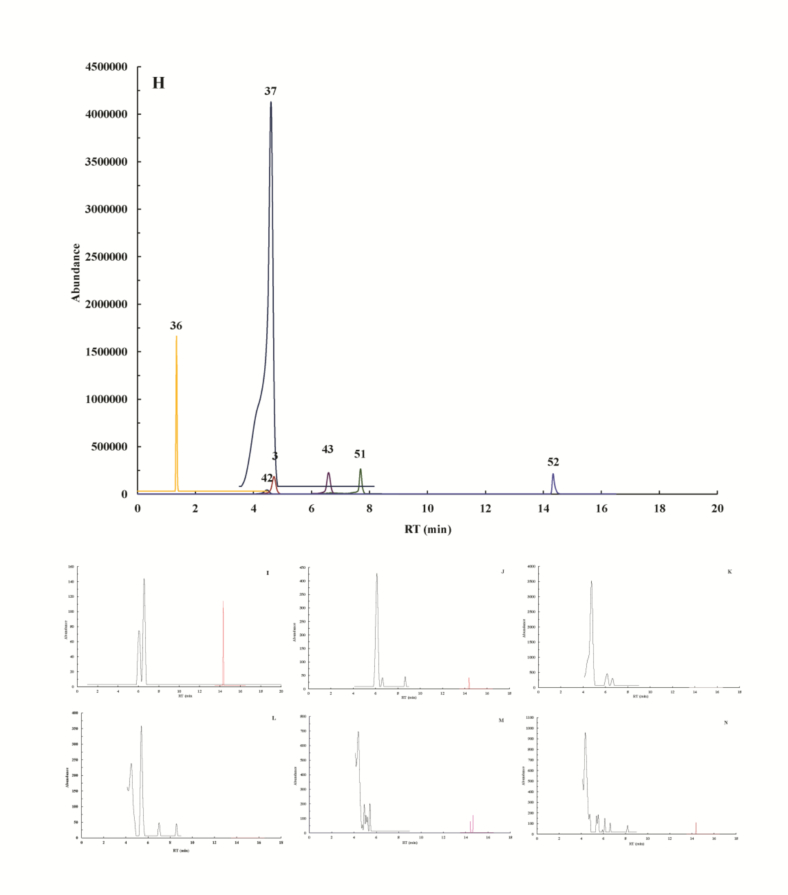
Extracted ion chromatogram (EIC) of 7 phenolic compounds of standard substance (H), free phenolic fractions of NL (I), TL (J) and FL (K), bound phenolic fractions of NL (L), TL (M) and FL (N) on negative mode. Compounds are numbered based on retention times (RT) and the corresponding names are given in Supplementary documents Table S 2.

Fig. 4 and Fig. 5 displayed the extracted ion chromatograms (EIC) of 63 phenolic compounds analyzed by UPLC-MS, including both standard substances and FP and BP from *F. hirta* Vahl. root cortex, acquired under positive and negative ionization modes‌. Quantitative analysis of *F. hirta* Vahl. root cortex revealed 57 phenolic compounds in free and bound fractions of three principal classes: 15 phenolic acids, 30 flavonoids, and 12 lignans and coumarins (Table 1).

**Table 1:** The identified phenolic compounds from free and bound phenolic fractions of *F. hirta* Vahl. root cortex of NL, TL and FL based on the UHPLC-MS/MS method.

Class	Name	Fraction	Content (μg/g)		
**Phenolic acid**			NL	TL	FL
1	Gallic acid	FP	19.35 ± 0.00a	19.34 ± 0.00a	19.35 ± 0.02a
		BP	9.69 ± 0.02a	9.71 ± 0.03a	9.68 ± 0.01a
2	Coumalic acid	FP	14.51 ± 0.00a	14.48 ± 0.09a	14.90 ± 0.49a
		BP	7.45 ± 0.00a	8.11 ± 0.87a	8.67 ± 1.22a
3	Neochlorogenic acid	FP	20.27 ± 0.02b	20.26 ± 0.01b	20.62 ± 0.12a
		BP	10.13 ± 0.00a	10.13 ± 0.00a	10.13 ± 0.00a
4	Protocatechuic acid	FP	8.77 ± 0.05b	9.07 ± 0.03a	8.80 ± 0.03b
		BP	4.41 ± 0.03b	5.13 ± 0.30a	4.43 ± 0.07b
5	Chlorogenic acid	FP	11.15 ± 0.04c	22.59 ± 0.73b	291.75 ± 5.28a
		BP	ND	5.31 ± 0.00a	5.31 ± 0.00a
6	Cryptochlorogenic acid	FP	12.12 ± 0.08b	23.34 ± 1.58b	305.31 ± 15.82a
		BP	5.79 ± 0.00a	5.79 ± 0.00a	5.79 ± 0.00a
7	Syringic acid	FP	2.81 ± 0.01b	2.94 ± 0.03a	2.77 ± 0.01c
		BP	1.36 ± 0.00a	1.37 ± 0.00a	1.37 ± 0.00a
8	Vanillic acid	FP	4.65 ± 0.19b	6.27 ± 0.21a	3.00 ± 0.10c
		BP	1.18 ± 0.07b	1.35 ± 0.00a	1.30 ± 0.07ab
9	Caffeic acid	FP	ND	ND	ND
		BP	6.23 ± 3.36b	15.63 ± 6.39a	10.26 ± 3.20ab
10	3-O-*p*-Coumaroylquinic acid	FP	18.04 ± 0.00b	18.04 ± 0.00b	20.85 ± 0.45a
		BP	ND	ND	9.02 ± 0.00
11	Ferulic Acid	FP	5.08 ± 0.00a	5.08 ± 0.00a	5.09 ± 0.00a
		BP	139.31 ± 2.85a	145.41 ± 4.02a	137.17 ± 5.13a
12	3-Hydroxycinnamic Acid	FP	3.76 ± 0.01a	3.75 ± 0.00a	3.76 ± 0.01a
		BP	2.02 ± 0.06a	2.00 ± 0.03ab	1.92 ± 0.05b
13	3,4-dihydroxybenzaldehyde	FP	8.31 ± 0.00ab	8.34 ± 0.03a	8.30 ± 0.00b
		BP	4.23 ± 0.03ab	4.26 ± 0.02a	4.21 ± 0.02b
14	Cinnamic acid	FP	4.22 ± 0.00a	4.23 ± 0.00a	4.22 ± 0.00a
		BP	2.10 ± 0.00a	2.11 ± 0.01a	2.11 ± 0.00a
15	Coniferin	FP	422.19 ± 81.60a	77.63 ± 12.16c	205.00 ± 12.78b
		BP	555.41 ± 82.75a	460.98 ± 32a	345.56 ± 87.60a
**Flavonoids**					
1	Procyanidin B1	FP	20.99 ± 0.20b	21.26 ± 0.29b	32.52 ± 0.85a
		BP	10.00 ± 0.00a	10.01 ± 0.00a	10.01 ± 0.01a
2	Catechin	FP	9.06 ± 0.01b	9.12 ± 0.02b	14.45 ± 0.38a
		BP	4.53 ± 0.00a	ND	4.54 ± 0.00a
3	Epicatechin	FP	8.70 ± 0.00b	8.79 ± 0.02b	14.57 ± 0.52a
		BP	4.36 ± 0.02a	4.40 ± 0.01a	4.34 ± 0.02a
4	Procyanidin B2	FP	17.64 ± 0.28c	19.58 ± 0.36b	21.15 ± 0.31a
		BP	ND	8.59 ± 0.01a	8.59 ± 0.00a
5	Epigallocatechin gallate	FP	10.06 ± 0.00	ND	ND
		BP	ND	5.03 ± 0.00	ND
6	Gallocatechin gallate	FP	16.03 ± 0.00	ND	ND
		BP	ND	ND	ND
7	Schaftoside	FP	12.27 ± 0.16b	12.42 ± 0.07ab	12.74 ± 0.09a
		BP	5.92 ± 0.00a	5.92 ± 0.00a	5.92 ± 0.00a
8	Isoorientin	FP	16.75 ± 0.00a	16.75 ± 0.00a	16.75 ± 0.00a
		BP	ND	ND	ND
9	Epiafzelechin	FP	6.64 ± 0.01b	6.84 ± 0.04a	6.79 ± 0.03a
		BP	3.30 ± 0.00a	3.30 ± 0.00a	3.30 ± 0.00a
10	Quercetin	FP	12.15 ± 0.00a	ND	12.15 ± 0.00a
		BP	6.08 ± 0.01a	6.08 ± 0.00a	6.07 ± 0.00a
11	Rutin hydrate	FP	5.48 ± 0.00a	5.48 ± 0.00a	5.48 ± 0.00a
		BP	ND	2.74 ± 0.00	ND
12	Isovitexin	FP	5.67 ± 0.00b	5.67 ± 0.00b	5.70 ± 0.00a
		BP	2.83 ± 0.00a	2.83 ± 0.00a	2.83 ± 0.00a
13	Vitexin	FP	11.71 ± 0.00b	11.72 ± 0.01b	11.79 ± 0.01a
		BP	ND	5.85 ± 0.00a	5.85 ± 0.00a
14	Rutin	FP	13.80 ± 0.00a	13.80 ± 0.00a	13.80 ± 0.00a
		BP	ND	ND	ND
15	Epicatechin gallate	FP	ND	ND	ND
		BP	2.58 ± 0.00a	2.58 ± 0.00a	2.58 ± 0.00a
16	Catechin gallate	FP	ND	ND	ND
		BP	ND	2.33 ± 0.00a	2.33 ± 0.00a
17	Taxifolin	FP	9.13 ± 0.00b	9.13 ± 0.00b	9.15 ± 0.01a
		BP	4.56 ± 0.00a	4.56 ± 0.00a	4.56 ± 0.00a
18	Kaempferol-3-rutinoside	FP	19.81 ± 0.00	ND	ND
		BP	ND	ND	ND
19	Aromadendrin	FP	4.37 ± 0.00a	4.37 ± 0.00a	4.37 ± 0.00a
		BP	2.19 ± 0.00a	2.19 ± 0.00a	2.19 ± 0.00a
20	Eriodictyol	FP	ND	0.06 ± 0.00b	0.68 ± 0.12a
		BP	ND	0.04 ± 0.00a	0.03 ± 0.00a
21	Luteolin	FP	3.34 ± 0.00a	3.34 ± 0.00a	3.34 ± 0.00a
		BP	1.67 ± 0.00a	1.67 ± 0.00a	1.67 ± 0.00a
22	Apigenin	FP	0.27 ± 0.00b	0.27 ± 0.00b	0.30 ± 0.01a
		BP	0.14 ± 0.00a	0.14 ± 0.00a	0.14 ± 0.00a
23	Phloretin	FP	0.02 ± 0.00a	0.02 ± 0.00a	0.02 ± 0.00a
		BP	0.01 ± 0.00a	0.01 ± 0.00a	0.01 ± 0.00a
24	Tectorigenin	FP	0.31 ± 0.00a	0.31 ± 0.00a	0.31 ± 0.00a
		BP	0.15 ± 0.00a	0.15 ± 0.00a	0.15 ± 0.00a
25	Diosmetin	FP	0.14 ± 0.00a	0.14 ± 0.00a	0.14 ± 0.00a
		BP	0.07 ± 0.00a	0.07 ± 0.00a	0.07 ± 0.00a
26	Kaempferol	FP	8.04 ± 0.00a	8.05 ± 0.00a	8.05 ± 0.00a
		BP	4.03 ± 0.00a	4.03 ± 0.00a	4.05 ± 0.03a
27	Isorhamnetin	FP	4.11 ± 0.00a	4.11 ± 0.00a	4.11 ± 0.00a
		BP	2.06 ± 0.00a	2.06 ± 0.00a	2.06 ± 0.00a
28	Afzelechin	FP	ND	ND	ND
		BP	261.00 ± 54.21a	230.76 ± 134.7a	199.35 ± 24.35a
29	Naringenin	FP	ND	ND	ND
		BP	292.71 ± 58.23a	254.35 ± 145.02a	229.38 ± 25.06a
30	Kempferide	FP	3.21 ± 0.00a	3.21 ± 0.00a	ND
		BP	ND	1.61 ± 0.00a	1.61 ± 0.00a
**Lignans and Coumarins**					
1	Esculin	FP	5.68 ± 0.00b	5.68 ± 0.00b	5.69 ± 0.00a
		BP	2.84 ± 0.00a	2.84 ± 0.00a	2.84 ± 0.00a
2	Esculetin	FP	4.03 ± 0.00b	4.04 ± 0.00b	4.06 ± 0.01a
		BP	2.02 ± 0.00a	2.03 ± 0.01a	2.03 ± 0.01a
3	Fraxetin	FP	1.76 ± 0.03a	1.74 ± 0.00a	1.74 ± 0.00a
		BP	0.90 ± 0.04a	0.90 ± 0.03a	0.89 ± 0.02a
4	Umbelliferone	FP	13.52 ± 0.94c	25.16 ± 1.97b	67.90 ± 1.98a
		BP	104.52 ± 9.54a	99.76 ± 9.00a	107.40 ± 5.70a
5	Marmesin	FP	4.19 ± 0.26b	6.05 ± 0.40a	6.58 ± 0.20a
		BP	0.13 ± 0.00a	0.13 ± 0.00a	0.13 ± 0.00a
6	Columbianetin	FP	0.16 ± 0.00a	0.16 ± 0.00a	0.16 ± 0.00a
		BP	0.08 ± 0.00a	0.08 ± 0.00a	0.08 ± 0.00a
7	Psoralen	FP	12,249.71 ± 89.57c	16,593.52 ± 55.88b	17,849.79 ± 334.22a
		BP	8.28 ± 1.00c	19.26 ± 2.05b	50.36 ± 3.60a
8	Sphondin	FP	0.14 ± 0.00a	0.14 ± 0.00a	0.14 ± 0.00a
		BP	0.09 ± 0.01b	0.08 ± 0.00b	0.10 ± 0.00a
9	Bergapten	FP	206.58 ± 1.44b	405.00 ± 1.74a	449.52 ± 35.67a
		BP	0.21 ± 0.01c	0.35 ± 0.02b	0.62 ± 0.06a
10	Demethylsuberosin	FP	0.31 ± 0.00b	0.65 ± 0.03b	1.51 ± 0.27a
		BP	0.14 ± 0.00a	0.14 ± 0.00a	0.14 ± 0.00a
11	Sesamin	FP	17.28 ± 4.58b	42.96 ± 6.79a	9.18 ± 1.17c
		BP	ND	ND	ND
12	Cnidioside	FP	0.22 ± 0.00a	ND	0.22 ± 0.00a
		BP	0.11 ± 0.00a	0.11 ± 0.00a	0.11 ± 0.00a

“ND” in the table means that the corresponding compound was not detected. Different lowercase letters (a-c) ni the same row indicated statistically significant differences between different leaf morphotypes (*p* < 0.05).

**Table S 1.**

Gallic acid, coumalic acid, neochlorogenic acid, protocatechuic acid, chlorogenic acid, cryptochlorogenic acid, syringic acid, vanillic acid, caffeic acid, 3-O-*p*-coumaroylquinic acid, ferulic acid, 3-hydroxycinnamic acid, 3,4-dihydroxybenzaldehyde, cinnamic acid and coniferin were all detected in both free and bound fractions of the *F. hirta* Vahl. root cortex in each morphotype. There were no significant differences among morphotypes for the free phenolic concentration of gallic acid, coumalic acid, ferulic acid, 3-hydroxycinnamic acid, or cinnamic acid (*p* *>* *0.05*). However, syringic acid, vanillic acid and coniferin exhibited distinct variations among morphotypes (*p* *<* *0.05*).

Chlorogenic acid, cryptochlorogenic acid and coniferin were the main phenolics in *F. hirta* Vahl. root cortex. Chlorogenic acid (291.75 ± 5.28 μg/g) and cryptochlorogenic acid (305.31 ± 15.82 μg/g) was present at higher concentrations in FL compared to NL and TL. Notably, content of coniferin in NL (422.19 ± 81.60 μg/g) significantly exceeded that in TL and FL (*p* *<* *0.05*). In BP, only protocatechuic acid, vanillic acid, 3-hydroxycinnamic acid and 3,4-dihydroxybenzaldehyde, displayed significant differences among the three leaf morphotypes (*p* *>* *0.05*). TL exhibited higher concentrations of protocatechuic acid (5.13 ± 0.30 μg/g), vanillic acid (1.35 ± 0.00 μg/g) and 3,4-dihydroxybenzaldehyde (4.26 ± 0.02 μg/g) comparing to NL and FL (*p* *<* *0.05*). Coniferin remained the predominant phenolic acid in BP, displaying concentration of NL, TL and FL were 555.41 ± 82.75, 460.98 ± 32 and 345.56 ± 87.60 μg/g, respectively.

30 different flavonoids were identified. Epicatechin gallate, catechin gallate, afzelechin, and naringenin were detected only as bound phenolics. Likewise, gallocatechin gallate, isoorientin, rutin, and kaempferol-3-O-rutinoside were present only in the FP fraction. There were some patterns of accumulation specific to morphotype. For example, procyanidin B1 (32.52 ± 0.85 μg/g), catechin (14.45 ± 0.38 μg/g), epicatechin (14.57 ± 0.52 μg/g), and procyanidin B2 (21.15 ± 0.31 μg/g) were significantly higher in the FL morphotype than in either NL or TL (*p* *<* *0.05*). Interestingly, afzelechin and naringenin were only identified in the bound form in *F. hirta* Vahl. root cortex, and their content were significantly higher than other flavonoid compounds.

12 lignan and coumarin compounds were identified, and all except sesamin appeared in both free and bound forms. Within the free phenolic fraction, psoralen was consistently the predominant component across all three morphotypes, with concentrations of 12,249.71 ± 89.57 μg/g in NL, 16,593.52 ± 55.88 μg/g in TL, and 17,849.79 ± 334.22 μg/g in FL. Psoralen content increased significantly with number of leaf lobes (*p* *<* *0.05*), indicating a morphotype-associated pattern. Bergapten was the second most abundant free phenolic and likewise exhibited higher levels in FL (449.52 ± 35.67 μg/g) than in NL (206.58 ± 1.44 μg/g) and TL (405.00 ± 1.74 μg/g). Furthermore, free umbelliferone and sesamin also differed significantly among morphotypes (*p* *<* *0.05*), although only psoralen and bergapten showed consistently higher levels in FL relative to both NL, TL. In contrast, umbelliferone was main bound component, but its concentrations 104.52 ± 9.54 μg/g in NL, 99.76 ± 9.00 μg/g in TL, and 107.40 ± 5.70 μg/g in FL, did not differ among leaf types (*p* *>* *0.05*). These patterns suggested that morphotype-related variation primarily affects free rather than bound lignan and coumarin accumulation.

Phenotype is ultimately a product of both genotype and growth environment, and leaf phenotypic variation may reflect genetic variation (Yang & Zhang, 2006; Yang et al., 2016; Chen et al., 2019; Raudone et al., 2024). Yan et al. (2025) randomly selected 100 EST-SSR primers to amplify target products using genomic DNA from four different leaf morphotypes of *F. hirta* Vahl., and 89 of these primers exhibited polymorphism across them, indicating functional gene region variations among the four leaf morphotypes. Most medicinal plant intraspecific variations are widespread, with different intraspecific variation groups generating distinct chemical compositions and quantities through differential expression of genes related to secondary metabolite synthesis. This has the ultimate consequence that different phenotypes become associated with varying medicinal quality levels (Zhang, 2011). For example, studies relate significant differences in the contents of atractylodin, atractylenolide I, β-eudesmol, and atractylon to four different leaf morphotypes of *A. chinensis* (Liu et al., 2024). In *Rosmarinus officinalis* L., the flavonoid content in *R. officinalis* ‘Lockwood de Forest’ was lower than in *R. officinalis* ‘Severn Sea’ and *R. officinalis* ‘Albus’(Ma, 2022). Secondary metabolites like alkaloids vary among different leaf morphotypes in *Pinellia ternata*. (Yang, 2013).

Similarly, Chen, Wang, and Li (2021) has already observed differences in total polyphenols and polyphenol oxidase activity in *F. hirta* Vahl. with different leaf morphotypes, with weaker polyphenol oxidase activity and lower polyphenol content in *F. hirta* Vahl. with FL. However, the results of this study indicated that not only the total polyphenol content in FL exceed that of the other two leaf types, but also several phenolic compounds, including chlorogenic acid, cryptochlorogenic acid, and psoralen. These differences may be attributed to variations in sample type, harvesting time, and growth environment. Likewise, any differences observed here in phenolic compounds among different leaf morphotypes of *F. hirta* Vahl. may be attributed to genotype, and further verification studies are required on the related genes.

In terms of the composition and content of polyphenols, *F. hirta* Vahl. root cortex is a good source of polyphenolic compounds compared to other plants. As reported by Kar et al. (2023), 11 phenolic compounds were detected in the ethanol extract of *Ficus religiosa* leaves, with quinic acid identified as the most abundant component at a concentration of 5.16 mg/g. In contrast, 57 phenolic compounds were identified in the root cortex of *F. hirta* Vahl., where psoralen represented the dominant constituent, with its content ranging from 12.24 to 17.85 mg/g. Notably, the polyphenol composition of *F. hirta* Vahl. root cortex was comparable to that of the leaves and fresh fruits of *Ficus carica* L., as both were rich in flavonoids, phenolic acids and furanocoumarins (Hosseini et al., 2025). Although, Yu et al. (2020) developed an efficient and sustainable extraction protocol achieving a psoralen yield of 15.65 mg/g from *Ficus carica* L. leaves, this value was comparable to the psoralen content detected in *F. hirta* Vahl. root cortex.

### 3.4 Antioxidant activities of FP and BP *in vitro*

Oxidative damage has been considered as an important factor for aging and many chronic diseases (Saravanakumar et al., 2019). When free radicals are generated in excess, they can initiate radical chain reactions with proteins, lipids, DNA and other biomolecules, and cause damage to tissue (Zheng et al., 2022). We are actively seeking antioxidant crops or herbs with high scavenging capacity for free radical to be used for the treatment of inflammation and prophylactic disease prevention. Three different free radicals including DPPH·, ABTS· and OH· were selected to estimate the radical scavenging capacity of FP and BP from *F. hirta* Vahl. root cortex with NL, TL and FL. IC_50_ of DPPH·, ABTS· and OH· scavenging ability in different samples were presented in Table 2.

**Table 2:** IC_50_ of free and bound phenolic fraction from *F. hirta* Vahl. root cortex with NL, TL and FL.

IC_50_(mg/mL)	Fraction	NL	TL	FL	Vc(μg/mL)
ABTS radical	FP	0.47 ± 0.03b	0.44 ± 0.06b	0.62 ± 0.06a	12.71 ± 0.17
	BP	0.17 ± 0.02a	0.14 ± 0.02a	0.18 ± 0.03a	
DPPH radical	FP	0.14 ± 0.00a	0.13 ± 0.01ab	0.12 ± 0.01b	4.81 ± 0.17
	BP	0.16 ± 0.02a	0.13 ± 0.01a	0.15 ± 0.01a	
OH radical	FP	7.31 ± 0.84c	9.38 ± 0.15b	11.67 ± 0.01a	241.26 ± 12.81
	BP	7.46 ± 0.09b	7.77 ± 0.45ab	7.82 ± 0.22a	

In terms of FP, IC_50_ of ABTS· of FL was 0.62 ± 0.06 mg/mL, higher than the NL (0.47 ± 0.03 mg/mL) or TL (0.44 ± 0.06 mg/mL) morphotypes (*p* *<* *0.05*). However, IC_50_ of DPPH· of FL was only 0.12 ± 0.01 mg/mL, significantly lower than the NL morphotype (0.14 ± 0.00 mg/mL) (*p* *<* *0.05*). The IC_50_ of ABTS· and DPPH· of bound phenol content did not show vary among morphotypes (*p* *>* *0.05*). IC_50_ of OH· were higher than DPPH· and ABTS· both in FP and BP. And IC_50_ of OH· of FL was higher than that of NL and TL (*p* *<* *0.05*).

Generally speaking, the lower the IC_50_ value, the stronger the antioxidant. The IC_50_ of all sample extracts were higher than synthetic antioxidant Vc, suggesting *F. hirta* Vahl. root is rich in abundant promising natural antioxidants that retard aging and inflammation (Cheng, Yi, Chen, et al., 2017; Quan et al., 2022; Xiao et al., 2022; Ye et al., 2020). Compared to hairy fig fruit, *F. hirta* Vahl. root cortex showed stronger antioxidant activities *in vitro* (Chen et al., 2020). These may be attributed to the higher abundance of phenolic compounds in the *F. hirta* Vahl. root cortex. In the present study, all morphotypes exhibited significant antioxidant activity and high levels of phenolic compounds, making them excellent raw materials for food additives.

### 3.5 Identification of free and bound phenolic compounds composition across different leaf morphotypes based on PCA, HCA and correlation analysis

**Fig. 6:**
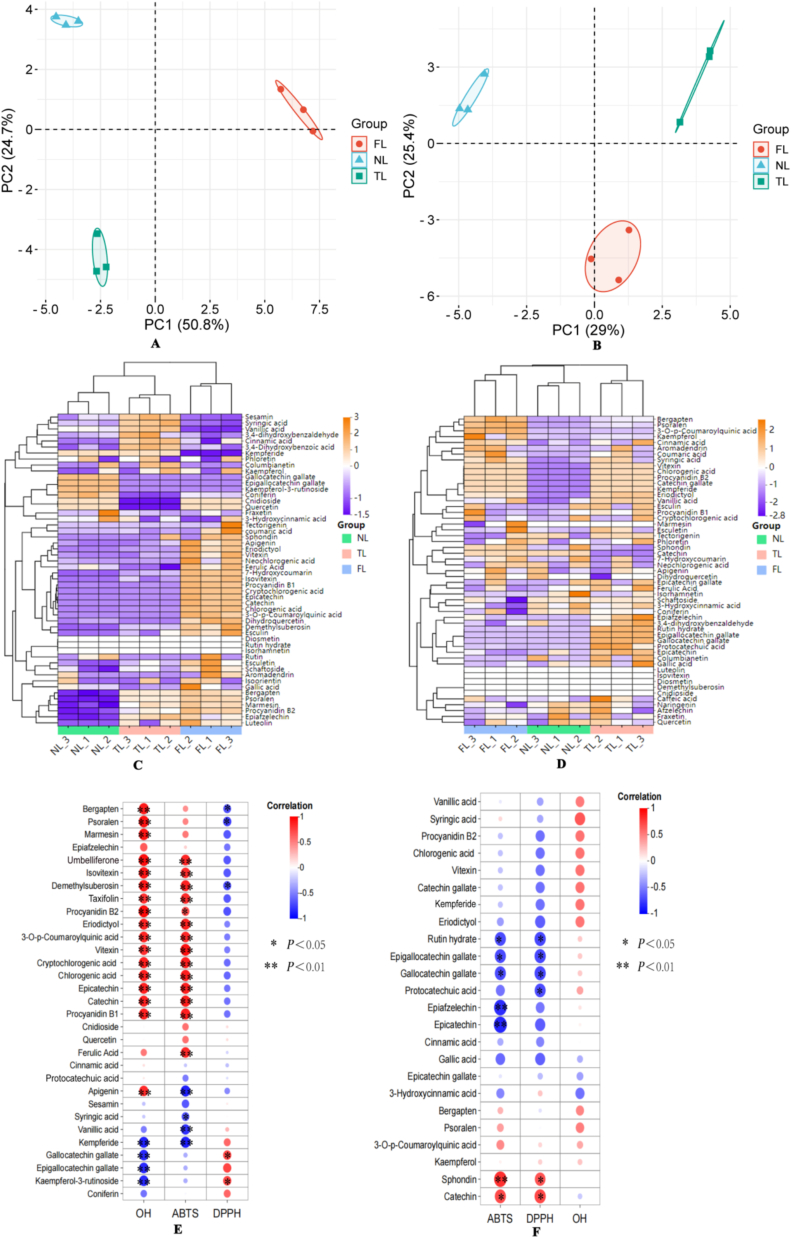
Principal component analysis, hierarchical cluster analysis and correlation analysis of free and bound phenolics profile. A, the scores biplot of PCA analysis of FP. B, the scores biplot of PCA analysis of BP. C, the heatmap of HCA analysis of FP. D, the heatmap of HCA analysis of BP. E, Correlation of free phenolic compounds and antioxidant capacity (ABTS•, DPPH•, OH•). F, Correlation of bound phenolic compounds and antioxidant capacity (ABTS•, DPPH•, OH•).*: *P* < 0.05, **; *P* < 0.01.

A bioplot of principal component analysis (PCA) on the free and bound phenolic compounds distribution of three leaf morphotypes of *F. hirta* Vahl. was shown in Fig. 6 A-B. For free phenolic fraction, PC1 explained 50.8% of the total variances and primarily represented a gradient from high furanocoumarin and acylquinic acid accumulation (e.g., psoralen, bergapten, chlorogenic acid, cryptochlorogenic acid) to higher levels of catechin-derived flavan-3-ols and related metabolites (e.g., epigallocatechin gallate, gallocatechin gallate, quercetin, coniferin) (Fig. 6 A). Specifically, FL showed a strong correlation with the furanocoumarin/acylquinic-acid end of PC1, implying a tendency toward the enrichment of psoralen, bergapten and related compounds. In contrast, NL was predominantly clustered in the catechin-dominated end of the same axis, while TL occupied an intermediate position by relatively higher contributions from protocatechuic acid, syringic acid and related simple phenolic acids. PC2 (24.7%) further captured differences between profiles enriched in simple phenolic acids versus more complex coumarins and flavonoids (Fig. 6 A). A similar pattern was observed in the bound phenolic fraction, where two components explained 54.4% of the total variance (Fig. 6 B). PC1 captured a gradient from highly bound furanocoumarins and coumarins (e.g., psoralen, bergapten, umbelliferone) to bound flavan-3-ols and hydroxybenzoic acids (e.g., epicatechin, rutin hydrate, gallic acid). FL again enriched in the furanocoumarins, whereas NL and TL dominated by flavan-3-ol/phenolic-acids, with TL showing relatively stronger associations with epicatechin-related constituents. These indicated that there were differences in free and bound phenolic compounds distribution of three leaf morphotypes, rather than differences in total phenolic abundance alone. To validate these PCA-derived metabolic patterns with an independent classification method, hierarchical clustering analysis (HCA) was subsequently performed.

Unlike PCA, HCA does not impose assumptions about cluster structure; instead, samples group exclusively according to their abundance patterns of target phenolic constituents. Consistent with this unbiased approach, samples belonging to the same morphotype clustered together in both free and bound phenolic datasets (Fig. 6 C—D), supporting the idea that morphotype-specific chemical organization is a genuine feature of *F. hirta* Vahl. root cortex. Importantly, the structure of the resulting clusters provided insight into the biochemical basis of morphotype differentiation. The clustering patterns largely mirrored those identified by PCA, with free phenolics showing stronger separation. The agreement between PCA and HCA confirmed that the major axes of variation were driven by coordinated shifts in free phenolics rather than random noise. Taken together, the PCA and HCA results converged on the same conclusion: the primary chemical distinctions among NL, TL, and FL arise from coordinated changes in free phenolics, whereas bound phenolics showed comparatively limited morphotype-specific variation. Similar phenotype-associated differences in the distribution of phenolic constituents had been reported in other species, such as *Lithocarpus litseifolius*, where free phenolics varied strongly across leaf forms, whereas bound phenolics were comparatively stable (Wang et al., 2022).

Moreover, correlation analysis was performed to determine which phenolic compounds most strongly contributed to antioxidant activity. As we know, polyphenols showed a very high correlation with antioxidant activity. Pearson's correlation coefficients between free and bound phenolic compounds and antioxidant activities present in *F. hirta* Vahl. root cortex with NL, TL and FL were shown in Fig. 6. In the FP (Fig. 6 E), IC_50_ of OH• was strongly correlated with epigallocatechin gallate, gallocatechin gallate, kaempferol-3-rutinoside and kempferide content (*p* *<* *0.01*). IC_50_ of ABTS• had significant associations with vanillic acid, kempferide and apigenin (*p* *<* *0.01*). IC_50_ of DPPH• was correlated with with psoralen, bergapten, demethylsuberosin content (*p* *<* *0.05*). In BP (Fig. 6 F), IC_50_ of ABTS• were driven by epicatechin and epiafzelechin (*p* *<* *0.01*), epigallocatechin gallate, gallocatechin gallate and rutin hydrate (*p* *<* *0.05*). IC_50_ of DPPH• were regulated by epigallocatechin gallate, gallocatechin gallate, rutin hydrate and protocatechuic acid (*p* < *0.05*). And IC_50_ of OH• had no significant correlations detected (*p* *>* *0.05*). From Fig. 6 E and Fig. 6 F, psoralen, bergapten, epigallocatechin gallate, gallocatechin gallate, kaempferol-3-rutinoside, kempferide, vanillic acid, apigenin and demethylsuberosin were key phenolics to explain the antioxidant activities of FP, and epicatechin, epiafzelechin, epigallocatechin gallate, gallocatechin gallate, rutin hydrate and protocatechuic acid were key compounds to explain the antioxidant activities of BP. Psoralen is a type of furan coumarin compounds derived from *Psoralea corylifolia* L., and is widely present in traditional Chinese medicine such as *Glehnia littoralis* Fr. Schmidt ex Miq., *Saposhnikovia divaricata* (Turcz.) Schischk, and *Angelica pubescens* (Bai et al., 2020; Li et al., 2019; Zhou & Zeng, 2019). It has pharmacological activities such as anti-tumor, neuroprotective, anti-inflammatory, and antioxidant effects, and can be used to treat diseases such as rheumatoid arthritis, leukemia, and Alzheimer's disease. Psoralen is one of the quality evaluation indicators for medicinal materials, while its content showing variations between different sources and plants (Liu & Song, 2013; Ma et al., 2016). Among these phenolic compounds, psoralen content was the highest in this study, indicating it was the most important phenolic in *F. hirta* Vahl. root cortex. And it will be the key and characteristic phenol to distinguish different varieties.

## 4 Conclusion

This study indicated that *F. hirta* Vahl. root cortex is a rich source of free and bound phenolic compounds. 57 phenolics were detected in *F. hirta* Vahl. root cortex, and 49 phenolic compounds were quantified for the first time. PCA and HCA analyses demonstrated that *F. hirta* Vahl. root cortex of the FL morphotype was distinct from NL and TL. The FP and BP of *F. hirta* Vahl. root cortex of all leaf morphotypes showed high antioxidant activities. Psoralen, bergapten, epigallocatechin gallate, gallocatechin gallate, kaempferol-3-rutinoside, kempferide, vanillic acid, apigenin and demethylsuberosin were key phenolics to explain the antioxidant activities of FP, and epicatechin, epiafzelechin, epigallocatechin gallate, gallocatechin gallate, rutin hydrate and protocatechuic acid were key compounds to explain the antioxidant activities of BP. *F. hirta* Vahl. root is a potent antioxidant source to produce functional foods in the future. Furthermore, *F. hirta* Vahl. varieties with five-lobed may be an important direction for breeding high-quality varieties rich in phenols and *in vitro* antioxidant activity in the future.

## CRediT authorship contribution statement

**Qing Gui:** Writing – original draft, Formal analysis, Data curation. **Qingmian Chen:** Methodology. **Yufeng Zhang:** Writing – review & editing. **Xiu Zeng:** Visualization, Software. **Shiyu Li:** Resources, Investigation. **Jianxiong Huang:** Validation, Project administration, Conceptualization. **Xiuquan Wang:** Supervision, Funding acquisition.

## Declaration of competing interest

The authors declare that they have no known competing financial interests or personal relationships that could have appeared to influence the work reported in this paper.

## Data Availability

Data will be made available on request.

## Appendix A Supplementary data

Supplementary material Image 1
