# Supplementary material for: Differential analysis of free and bound phenolic compounds and its antioxidant activity of Ficus hirta Vahl. root cortex with different leaf morphotypes based on metabolomics
Source: Food Chem X. 2026 Jan 25;34:103554. doi: 10.1016/j.fochx.2026.103554 (PMC12874614; doi:10.1016/j.fochx.2026.103554)
Supplement: Supplementary file 1 — Supplementary material [file mmc1.docx]

Table S 1 56 phenolic standard substance on positive mode

| **NO.** | **Name** | **RT (min)** | **Parents ion** | **Daughters ion** | **Mode** |
| --- | --- | --- | --- | --- | --- |
| 1 | Fraxetin | 5.3 | 209.0619 | 149.1333 | EI+ |
| 2 | Eriodictyol | 10.33 | 288.9004 | 153.0974 | EI+ |
| 4 | Aromadendrin | 8.95 | 288.9643 | 153.0969 | EI+ |
| 5 | Marmesin | 9.85 | 247.0581 | 175.1565 | EI+ |
| 6 | Procyanidin B1 | 2.79 | 578.9136 | 127.0751 | EI+ |
| 7 | Isovitexin | 6.72 | 432.9651 | 283.059 | EI+ |
| 8 | Demethylsuberosin | 14.67 | 231.0366 | 175.0747 | EI+ |
| 9 | Epiafzelechin | 5.98 | 274.9828 | 139.0537 | EI+ |
| 10 | Kaempferol | 11.67 | 286.9528 | 69.1599 | EI+ |
| 11 | Coniferin | 17.14 | 344.1379 | 88.2038 | EI+ |
| 12 | Sesamin | 15.87 | 353.9777 | 148.8096 | EI+ |
| 13 | 3-Hydroxycinnamic Acid | 7.83 | 165.0643 | 91.1458 | EI+ |
| 14 | Sphondin | 11.87 | 217.0304 | 161.1219 | EI+ |
| 15 | Procyanidin B2 | 4.06 | 578.9136 | 127.0751 | EI+ |
| 16 | Angelicin | 11.88 | 187.0643 | 131.17 | EI+ |
| 17 | Cryptochlorogenic acid | 3.78 | 354.9589 | 163.1121 | EI+ |
| 18 | Phloretin | 11.49 | 275.0466 | 107.1268 | EI+ |
| 19 | Shikimicacid | 11.81 | 174.4798 | 154.0844 | EI+ |
| 20 | Esculin | 2.79 | 340.9289 | 179.0818 | EI+ |
| 21 | Afzelechin | 14.16 | 274.0891 | 88.149 | EI+ |
| 22 | Ferulic Acid | 7.05 | 195.0204 | 145.1038 | EI+ |
| 23 | Vanillic acid | 4.52 | 168.9904 | 93.1043 | EI+ |
| 24 | Cinnamic acid | 11.11 | 149.0643 | 77.1637 | EI+ |
| 25 | Neochlorogenic acid | 1.38 | 354.9589 | 163.0488 | EI+ |
| 26 | Cnidioside | 16.56 | 245.1304 | 189.1081 | EI+ |
| 27 | Columbianetin | 10.33 | 247.1004 | 175.0791 | EI+ |
| 28 | Dihydroquercetin | 7.48 | 304.9628 | 153.0969 | EI+ |
| 29 | Esculetin | 4.34 | 179.0443 | 123.1375 | EI+ |
| 30 | Apigenin | 11.41 | 270.9528 | 153.0961 | EI+ |
| 31 | coumalic acid | 2.4 | 140.9943 | 95.0428 | EI+ |
| 32 | Diosmetin | 11.66 | 300.9728 | 286.0396 | EI+ |
| 33 | Epicatechin | 3.56 | 291.0466 | 139.1188 | EI+ |
| 34 | Epicatechin gallate | 7.19 | 442.9551 | 139.1097 | EI+ |
| 35 | Epigallocatechin gallate | 4.83 | 458.9551 | 139.1113 | EI+ |
| 38 | Catechin | 3.58 | 290.9828 | 139.1193 | EI+ |
| 39 | Catechin gallate | 7.25 | 443.0189 | 139.1094 | EI+ |
| 40 | Orientin | 5.91 | 448.9651 | 329.0493 | EI+ |
| 41 | Quercetin | 6.64 | 302.9528 | 153.1009 | EI+ |
| 44 | Chlorogenic acid | 3.51 | 355.0228 | 163.1099 | EI+ |
| 45 | Gallocatechin | 2.12 | 307.0466 | 151.0861 | EI+ |
| 46 | Gallocatechin gallate | 5.27 | 458.9551 | 139.1113 | EI+ |
| 47 | Gallic acid | 1.32 | 171.0243 | 109.0826 | EI+ |
| 48 | Vitexin | 6.8 | 433.0289 | 313.032 | EI+ |
| 49 | Luteolin | 10.34 | 287.0166 | 153.0999 | EI+ |
| 50 | Troxerutin | 7.25 | 742.9428 | 435.022 | EI+ |
| 53 | Schaftoside | 5.4 | 564.9474 | 121.0961 | EI+ |
| 54 | Isoorientin | 5.51 | 448.9651 | 299.0164 | EI+ |
| 55 | Isorhamnetin | 11.83 | 316.9089 | 153.0901 | EI+ |
| 56 | Naringenin | 14.16 | 274.1755 | 88.2037 | EI+ |
| 57 | Tectorigenin | 11.53 | 300.9089 | 286.0406 | EI+ |
| 58 | 3,4-dihydroxybenzaldehyde | 3.74 | 138.9604 | 65.1413 | EI+ |
| 59 | Protocatechuic acid | 2.67 | 154.9604 | 93.1122 | EI+ |
| 60 | Rutin hydrate | 6.64 | 610.9136 | 302.9917 | EI+ |
| 61 | Umbelliferone | 6.87 | 162.9804 | 107.1379 | EI+ |
| 62 | Psoralen | 11.58 | 187.0643 | 115.086 | EI+ |
| 63 | Bergapten | 12.82 | 217.0304 | 202.0267 | EI+ |

Table S 2 7 phenolic standard substance on negative mode

| **NO.** | **Name** | **RT (min)** | **Parents ion** | **Daughters ion** | **Mode** |
| --- | --- | --- | --- | --- | --- |
| 3 | 3-O-p-Coumaroylquinic acid | 4.84 | 336.9404 | 191.1305 | EI- |
| 36 | Epigallocatechin | 1.33 | 307.0466 | 139.1208 | EI- |
| 37 | syringic acid | 4.67 | 199.0743 | 140.1123 | EI- |
| 42 | Caffeic acid | 4.55 | 179.0004 | 135.7002 | EI- |
| 43 | Quercetin-3-O-rutinoside | 6.67 | 608.8498 | 300.072 | EI- |
| 51 | Kaempferol-3-rutinoside | 7.78 | 592.9136 | 284.7994 | EI- |
| 52 | Kempferide | 14.41 | 299.0366 | 284.0388 | EI- |
